# Neoadjuvant Chemoradiotherapy for Oral Cavity Cancer: Predictive Factors for Response and Interim Analysis of the Prospective INVERT-Trial

**DOI:** 10.3389/fonc.2022.817692

**Published:** 2022-03-24

**Authors:** Jens von der Grün, Ria Winkelmann, Iris Burck, Daniel Martin, Franz Rödel, Peter Johannes Wild, Katrin Bankov, Andreas Weigert, Ivan-Maximiliano Kur, Christian Brandts, Natalie Filmann, Christian Issing, Philipp Thönissen, Anna Maria Tanneberger, Claus Rödel, Shahram Ghanaati, Panagiotis Balermpas

**Affiliations:** ^1^ Department of Radiotherapy and Oncology, Goethe-University Frankfurt, Frankfurt, Germany; ^2^ German Cancer Research Center (DKFZ), Heidelberg, Germany; ^3^ German Cancer Consortium (DKTK), Partner Site Frankfurt a. M., Goethe-University Frankfurt, Frankfurt, Germany; ^4^ Frankfurt Cancer Institute (FCI), Goethe-University Frankfurt, Frankfurt, Germany; ^5^ Dr. Senckenberg Institute of Pathology, Goethe-University Frankfurt, Frankfurt, Germany; ^6^ Department of Diagnostic and Interventional Radiology, Goethe-University Frankfurt, Frankfurt, Germany; ^7^ Institute of Biochemistry I, Faculty of Medicine, Goethe-University Frankfurt, Frankfurt, Germany; ^8^ Department of Medicine, Hematology/Oncology, Goethe-University Frankfurt, Frankfurt, Germany; ^9^ Institute of Biostatistics and Mathematical Modelling, Goethe-University Frankfurt, Frankfurt, Germany; ^10^ Department of Otorhinolaryngology, Goethe-University Frankfurt, Frankfurt, Germany; ^11^ Department of Oral, Maxillofacial and Facial Plastic Surgery, Goethe-University Frankfurt, Frankfurt, Germany; ^12^ Department of Radiation Oncology, University Hospital Zurich, Zurich, Switzerland

**Keywords:** neoadjuvant chemoradiotherapy, oral cavity cancer, multiplexed immunofluorescence, diffusion-weighted magnetic resonance imaging, predictive biomarker

## Abstract

**Background:**

To study neoadjuvant chemoradiotherapy (nCRT) and potential predictive factors for response in locally advanced oral cavity cancer (LA-OCC).

**Methods:**

The INVERT trial is an ongoing single-center, prospective phase 2, proof-of-principle trial. Operable patients with stage III-IVA squamous cell carcinomas of the oral cavity were eligible and received nCRT consisting of 60 Gy with concomitant cisplatin and 5-fluorouracil. Surgery was scheduled 6-8 weeks after completion of nCRT. Explorative, multiplex immunohistochemistry (IHC) was performed on pretreatment tumor specimen, and diffusion-weighted magnetic resonance imaging (DW-MRI) was conducted prior to, during nCRT (day 15), and before surgery to identify potential predictive biomarkers and imaging features. Primary endpoint was the pathological complete response (pCR) rate.

**Results:**

Seventeen patients with stage IVA OCC were included in this interim analysis. All patients completed nCRT. One patient died from pneumonia 10 weeks after nCRT before surgery. Complete tumor resection (R0) was achieved in 16/17 patients, of whom 7 (41%, 95% CI: 18-67%) showed pCR. According to the Clavien-Dindo classification, grade 3a and 3b complications were found in 4 (25%) and 5 (31%) patients, respectively; grade 4-5 complications did not occur. Increased changes in the apparent diffusion coefficient signal intensities between MRI at day 15 of nCRT and before surgery were associated with better response (p=0.022). Higher abundances of programmed cell death protein 1 (PD1) positive cytotoxic T-cells (p=0.012), PD1+ macrophages (p=0.046), and cancer-associated fibroblasts (CAFs, p=0.036) were associated with incomplete response to nCRT.

**Conclusion:**

nCRT for LA-OCC followed by radical surgery is feasible and shows high response rates. Larger patient cohorts from randomized trials are needed to further investigate nCRT and predictive biomarkers such as changes in DW-MRI signal intensities, tumor infiltrating immune cells, and CAFs.

## 1 Introduction

The standard treatment for locally advanced oral cavity cancer (LA-OCC) is primary surgery followed by risk-adapted adjuvant radiotherapy/chemoradiotherapy (RT/CRT) or definitive CRT for functionally inoperable tumors (1–5). Following combined modality treatment, local recurrences and distant metastases occur in about 25% of patients with locally advanced head and neck squamous cell carcinoma (LA-HNSCC) (1, 2). However, local control rates for the subgroup of LA-OCC remain inferior to those of LA-HNSCC with most locoregional failures emerging in field of prior RT (6–8). Furthermore, high-dose, postoperative RT/CRT to the oral cavity is challenging following extensive reconstructive surgery and can be delayed due to prolonged postoperative recovery or possible complications associated with surgery (6, 9, 10). Also, better vascularization and oxygenation in the unoperated tissue is associated with increased radiosensitivity and early systemic therapy could potentially reduce metastatic spread of these tumors (11). Some rare complications, such as fibula transplant- or flap-necrosis related to RT could be avoided in case of preoperative treatment, and in case of occurrence, the necrotic jaw could be resected during surgery (9). To improve local tumor control and overcome some of the limitations of primary or postoperative radiotherapy (PORT), a limited number of retrospective and prospective studies investigated neoadjuvant RT/CRT in LA-OCC. These studies mostly showed encouraging local control rates despite utilizing partly outdated RT-techniques, doses, and time intervals between treatment modalities (12). To study neoadjuvant CRT (nCRT) we launched a prospective, single-arm trial investigating nCRT followed by surgery in LA-OCC. We here report on first results regarding feasibility and early efficacy with a particular focus on potential predictive biomarkers for pathologic complete response (pCR) based on pretreatment immune contextures and diffusion-weighted magnetic resonance imaging (DW-MRI) signal changes during treatment.

## 2 Patients And Methods

### 2.1 Patient Selection

The INVERT trial is an ongoing, single-center, prospective phase II trial. Eligible patients were 18 years or older with histologically confirmed, primary diagnosis of locally advanced HNSCC of the oral cavity stage III-IVA defined by UICC TNM version 8. Mandatory staging included MRI of the neck, and computed tomography (CT) of the chest and abdomen. Additional key inclusion criteria were Eastern Cooperative Oncology Group (ECOG) status of ≤2 and adequate organ function. The study received approval by the ethics committee of the Goethe-University Frankfurt, Frankfurt, Germany (approval number 208/12). A written informed consent was provided by each patient. The INVERT treatment schedule is shown in **Supplementary Figure 1**. The study protocol synopsis in English language is provided as **Supplementary Table 1**, the complete protocol in German language as supplementary document 1.

### 2.2 Chemoradiotherapy

Neoadjuvant RT consisted of 60.0/54.9/50.1 Gy in 30 fractions, applied to the primary tumor region, involved/high risk neck levels, and the elective neck levels according to current guidelines, respectively (13, 14). Intensity-modulated radiotherapy (IMRT) with a simultaneously integrated boost (SIB) concept was used. Therapy was delivered by 6 MeV photon energy using a linear accelerator (Versa HD™, Elekta). Two cycles of chemotherapy (CTX) were applied on days 1–5, and 29–33 of the RT consisting of 5-fluorouracil (5-FU) (600 mg/m² per day) as a continuous 120-h intravenous infusion, and cisplatin (20 mg/m² per day) as short intravenous infusion (15). For patients who were ineligible for cisplatin, carboplatin area und curve (AUC) 1 was applied alternatively on days 1–5, and 29–33. For patients with contraindications for 5-FU, cisplatin monotherapy was applied.

### 2.3 Surgery

Radical surgery following nCRT was performed according to the initial extension of the primary tumor as marked by pretreatment tattooing. Elective neck dissection was performed according to pretreatment staging information. Elective, ipsilateral supraomohyoid neck dissection (SOHND) was conducted for clinically negative neck nodes (cN0), and was extended to the neck levels I-V for pathologically positive nodes. In these cases, and for tumors crossing midline, contra-lateral SOHND was performed and also extended to the neck levels I-V for positive, contra-lateral nodes. Surgical reconstruction consisted of locoregional flaps, myocutaneous flaps, free flaps, or bone grafts as one- or two-stage surgical procedures.

### 2.4 Objectives

The primary endpoint, pCR, was defined as ypT0N0 after surgery. Acute and late adverse events from CRT and surgery were graded according to the National Cancer Institute Common Terminology Criteria for Adverse Events (NCI-CTCAE) version 4.0. Furthermore, surgical complications were graded on the basis of the Clavien-Dindo classification (16, 17). Explorative immune cell counts and DWI-MRI signal intensities were assessed to identify potential predictive bio- and imaging markers for pCR.

### 2.5 Pathological Assessment of Tumor Response

For pathological assessment, the tissue was extensively worked up. The tumor bed was formaldehyde-fixed and paraffin-embedded (FFPE) in total; ypTNM staging was applied according to the UICC TNM classification of malignant tumors (Union internationale contre le cancer, Version 8, 2017). Furthermore, tumor regression grading of the primary tumor was performed as described by Braun et al. (18): Grade 1: No or devitalized tumor cells; grade 2: small nests of vital tumor cells which do not exceed 5% of the whole lesion; grade 3: 5%-50% vital tumor cells; grade 4: more than 50% vital tumor cells. Also, for residual primary tumors, patterns of response to neoadjuvant CRT were evaluated as introduced by Nagtegaal et al. (19) and reported as tumor fragmentation versus shrinkage.

### 2.6 Radiological Assessment of Tumor Response

Diffusion-weighted, gadolinium enhanced MRI was performed prior to RT (day -14 to day 0; MRI 1), during RT (day 15, MRI 2), and prior to surgery (day 72 to 86, MRI 3).

#### 2.6.1 Magnetic Resonance Imaging Protocol

All MRI scans were performed using a 1.5-T system (MAGNETOM Avantofit, Siemens Healthineers) with a dedicated head and neck coil. Standard axial turbo inversion recovery magnitude (TIRM) (repetition time ms/echo time ms 3270/36; matrix size, 320 × 252; slice thickness, 6 mm), axial DW (diffusion-weighted) (repetition time ms/echo time ms, 3980/55; matrix size, 160 × 160; section thickness, 5 mm); axial unenhanced T1-weighted turbo spin-echo sequences (repetition time ms/echo time ms, 659/12; matrix size, 384 × 324; section thickness, 4 mm); axial T2-weighted turbo spin-echo sequences (repetition time ms/echo time ms, 7010/83; matrix size, 384 × 365; section thickness, 4 mm) were acquired. Axial contrast-enhanced T1-weighted multipoint Dixon sequences with fat suppression (repetition time ms/echo time ms, 604/12; matrix size, 320 × 277; section thickness, 4 mm) were also performed. Contrast administration was performed by injection of 0.1 ml gadobutrol per kilogram body weight (flow rate of 2 ml/s) with a power injector (Accutron MR; Medtron, Saarbrücken, Germany), followed by application of 20 ml saline (flow rate of 2 ml/s).

#### 2.6.2 Image Analysis

All MRI scans were analyzed on a commercially available PACS workstation (Centricity 4.2, GE Healthcare, Dornstadt, Germany). Two different observers (one radiology department resident, one senior staff member) quantitatively analyzed the MR series in consensus. Tumor signal intensities were assessed on diffusion-weighted, T2-weighted, and contrast-enhanced images using dedicated regions of interest (ROI) with a standardized radius of 5mm, placed on solid portions of the tumors. The signal intensity of the upper cervical spinal cord was also measured. The tumor signal intensities were expressed as a tumor to spine signal intensity ratio. Furthermore, ADC (apparent diffusion coefficient) were calculated with two b factors (0, 1,000 s/mm2) by placing ROIs over the solid tumor regions. Subsequently, the signal intensities of the tumors were independently assessed qualitatively by the two raters. The higher value was taken into account for the analysis in the event of unequal assessment by the two raters. Tumor signal intensities were evaluated on diffusion-weighted and T2-weighted images using a 5-point scale compared with the spinal cord (1 = hypointense, 2 = slightly hypointense, 3 = isointense, 4 = slightly hyperintense and 5 = markedly hyperintense). The images of the gadolinium-enhanced T1-weighted images were assessed using a 4-point scale compared to the submandibular gland (1 = no enhancement, 2 = weak enhancement, 3 = moderate enhancement, and 4 marked enhancement) (20).

### 2.7 Multiplexed Immunofluorescence

Pretreatment FFPE tissue sections were assessed before staining by an experienced head and neck pathologist. Each section contained the following three tumor compartments: tumor, invasive front, and tumor microenvironment (TME, stroma). Next, the pretreatment tissue sections (2 µm thick) were deparaffinized by 1 hour incubation at 60°C and stained with Opal 7‐Color Automation immunohistochemistry (IHC) Kits (Akoya Bioscience) in the BOND‐RX Multiplex IHC Stainer (Leica). Each section was put through 6 sequential rounds of staining, which included blocking in 5% BSA followed by incubation with primary antibodies of two panels (T-cell panel: CD3, Ventana, 790-4341; CD4, Abcam, ab133616; PD-1, Sigma, HPA035981-100UL; CD163, Abcam, ab182422; CD8, DAKO, M710301-2; FoxP3, Abcam, ab20034; TME panel: PD-L1, Spring, M4422; Pan-Cytokeratin (Pan-CK), Abcam, ab7753; alpha-smooth muscle actin (aSMA), Sigma, F377; Vimentin, Abcam, ab92547; CD45, Abcam, ab10558; Ki67, Abcam, ab16667), corresponding secondary HRP-conjugated antibodies (Akoya Biosciences, ARH1001A) and Opal fluorophores as described before (21). Nuclei were counterstained with 4′,6‐diamidino‐2‐phenylindole (DAPI) contained in the Opal 7‐Color Automation IHC Kits, and slides were mounted with Fluoromount‐G (SouthernBiotech). Imaging was performed with the VectraPolaris imaging system (Akoya Bioscience), and images were analyzed by using the Phenotyping application of the inForm software V2.5 (Akoya Bioscience). The following markers were used to identify specific cell types for input into the training algorithm: T-Helper Cells: CD3+ CD4+; Exhausted T-Helper Cells CD3+ CD4+ PD1+; Cytotoxic T-cells: CD3+ CD8+; Exhausted Cytotoxic T-Cells: CD3+ CD8+ PD1+; Macrophages: CD163+; PD1+ macrophages: CD163+ PD1+; Regulatory T-cells (Tregs): CD3+ CD4+ FoxP3+; Cancer-associated fibroblasts (CAFs): aSMA+ Vimentin+; PD-L1+ CAFs: aSMA+ Vimentin+ PD-L1+; Immune cells: CD45+; PD-L1+ immune cells: CD45+ PD-L1+. Proliferating immune cells: CD45+ Ki67+.

### 2.8 Statistics and Analysis

The primary clinical objective of this pilot study is to estimate the pCR rate and to calculate the corresponding 95% confidence interval. The assumed probability for pCR on which the case number calculation was based was 50%. In order for the overall statistical length to be less than 40% (+/- 20%), data from a total of n=26 patients must be available for analysis (exact Clopper-Pearson calculation using PASS 2008 software). Since the primary endpoint of pCR is achieved after surgery, we expect only a small drop out of at most 5%, resulting in a total number of 28 patients to be recruited.

Statistical analyses were performed using SPSS (IBM SPSS Statistics, v25.0, Armonk, NY, USA) and R [R Core Team (2020). R Foundation for Statistical Computing, Vienna, Austria]. Confidence intervals for binomial variables were calculated using the Clopper–Pearson method. Associations between categorical variables were evaluated by the Pearson chi-squared test. Regarding qualitative and quantitative MRI analysis, the Wilcoxon signed-rank test was used for nonparametric, related samples. Further, the Mann-Whitney U test was nonparametric, nonrelated samples for quantitative MRI analysis. Cohen’s Kappa test was used to assess the overall inter-rater variability in the qualitative MRI evaluation (22). For multiplexed immunofluorescence analysis, overall average marker percentages were dichotomized between “high” and “low” abundance by median value. All tests were two-sided and a p-value of p ≤ 0.05 was considered as significant during all statistical procedures.

## 3 Results

### 3.1 Patient Characteristics

Until the data cutoff for this interim analysis in July 2021, 17 of 26 planned patients were enrolled in this trial. All patients had stage IVA tumors of the oral cavity, mostly with osseus tumor infiltration (15/17, 88%); 59% (10/17) of the patients were men, and median age was 63 years by the time of first diagnosis. **Table 1** summarizes the patient characteristics. The consort diagram is shown in **Supplementary Figure 2**.

**Table 1:** Baseline characteristics. Clinical disease stage according to UICC TNM classification (8^th^ edition); ECOG, Eastern Cooperative Oncology Group.

Characteristic	n (%)
**Total**	17 (100)
**Sex**	
Male	10 (59)
Female	7 (41)
**Age**	
Median, years (range)	63 (42-76)
**ECOG performance status**	
0	14 (82)
1	3 (18)
**History of smoking**	
Yes	13 (76)
No	2 (12)
Missing	2 (12)
**History of alcohol abuse**	
Yes	7 (41)
No	8 (47)
Missing	2 (12)
**Tumor site**	
Oral cavity	17 (100)
**Clinical T category**	
cT1	0 (0)
cT2	2 (12)
cT3	0 (0)
cT4	15 (88)
**Clinical N category**	
cN0	2 (12)
cN1	1 (6)
cN2a	1 (6)
cN2b	11 (65)
cN2b	2 (12)
cN3	0 (0)
**Pathological tumor differentiation**	
Well differentiated (G1)	1 (6)
Moderately differentiated (G2)	16 (94)
Poorly differentiated (G3)	0 (0)
**Clinical disease stage**	
III	0 (0)
IVA	17 (100)

### 3.2 Toxicity, Treatment Compliance and Efficacy

RT-related grade 3 toxic effects occurred as pain and dysphagia in 4 (24%, 95% CI: 7-50%), as mucositis in 7 (41%, 95% CI: 18-67%), and as radiation dermatitis in 2 (12%, 95% CI: 1-36%) of the patients. Chemotherapy-related grade 3 adverse effects were leukopenia in 5 (29%, 95% CI: 10-56%), and hypertension in 6 (35%, 95% CI: 14-62%) patients. One patient with comorbidities died from pneumonia ten weeks after completion of nCRT (**Supplementary Table 2**).

Full dose of RT was applied in all 17 patients. Thirteen (76%, 95% CI: 50-93%) received cisplatin and 5-FU. Three patients (18%, 95% CI: 38-43%) with contraindications against 5-FU received cisplatin monotherapy, and one patient with contraindications for cisplatin received carboplatin and 5-FU. Regarding compliance with CTX, 13 (76%, 95% CI: 50-93%) patients completed CTX as prescribed and 4 (24%, 95% CI: 7-50%) received >50%. All patients received prophylactic gastric tubes (PEG tube) to ensure adequate nutrition (**Supplementary Table 3**).

After nCRT, 16 patients underwent surgery. All patients received bilateral neck dissections and flap plastics. Complete local tumor resection (R0) was achieved in all cases (100%, 95% CI: 79-100%). NCI-CTCAE grade 3 complications were reported in 9 (56%, 95% CI: 30-80%) cases. Oral hemorrhages (4/16, 25%, 95% CI: 7-52%) and wound complications (3/16, 19%, 95% CI: 4-46%) were most common. According to the Clavien-Dindo classification, grade 3a complications were found in 4 (25%, 95% CI: 7-52%) patients, and grade 3b complications were reported in 5 (31%, 95% CI: 11-59%) patients. Grade 4-5 surgical complications did not occur (**Table 2**).

**Table 2:** Surgical and pathological characteristics of patients who underwent surgery.

Characteristic	n (%)
**Total**	16 (100)
Time interval to surgery, days, median (range)	
From start of CRT to surgery	97 (69-121)
From end of CRT to surgery	56 (42-80)
**Surgery**	
Duration of surgery, minutes, median (range)	485 (369-802)
**Neck dissection**	
Ipsilateral	16 (100)
Contralateral	16 (100)
Number of dissected nodes, ipsilateral, median (range)	24 (11-60)
Number of dissected nodes, contralateral, median (range)	18 (5-39)
**Flap plastic**	
Regional	5 (31)
Vastus lateralis	4 (25)
Vastus lateralis and anterolateral thigh	2 (13)
Deltopectoral	2 (13)
Rectus abdominis	1 (6)
Radial forearm	1 (6)
Fibula	1 (6)
**Residual tumor**	
R0	16 (100)
R1/2	0 (0)
**Pathologic T category**	
ypT0	8 (50)
ypT1	4 (25)
ypT2	0 (0)
ypT3	0 (0)
ypT4	4 (25)
**Pathologic N category**	
ypN0	13 (81)
ypN1	2 (13)
ypN2a	0 (0)
ypN2b	1 (6)
ypN2c	0 (0)
ypN3	0 (0)
**Tumor regression grading^#^**	
1	8 (51)
2	6 (37)
3	1 (6)
4	1 (6)
**Primary tumor regression pattern**	
Tumor shrinkage	3 (19)
Tumor fragmentation	5 (31)
Pathologic complete response	8 (59)
**Postoperative morbidity**	
**Clavien-Dindo classification**	
None	5 (31)
Grade 1	1 (6)
Grade 2	1 (6)
Grade 3a	4 (25)
Grade 3b	5 (31)
Grade 4	0 (0)
Grade 5	0 (0)
NCI-CTCAE* complications	
None	5 (31)
Grade 1	1 (6)
Grade 2	1 (6)
Grade 3	9 (56)
Grade 4	0 (0)
Grade 5	0 (0)
NCI-CTCAE* complications grade ≥3	
Wound complication (including 1 loss of flap)	3 (19)
Oral hemorrhage	4 (25)
Hematoma	1 (6)
Laryngeal edema	1 (6)

*National Cancer Institute Common Terminology Criteria for Adverse Events version 5.0. ^#^Tumor regression of the primary tumor following neoadjuvant chemoradiotherapy according to Braun et al., 1989. CRT, Chemoradiotherapy.

**Figure 1:**
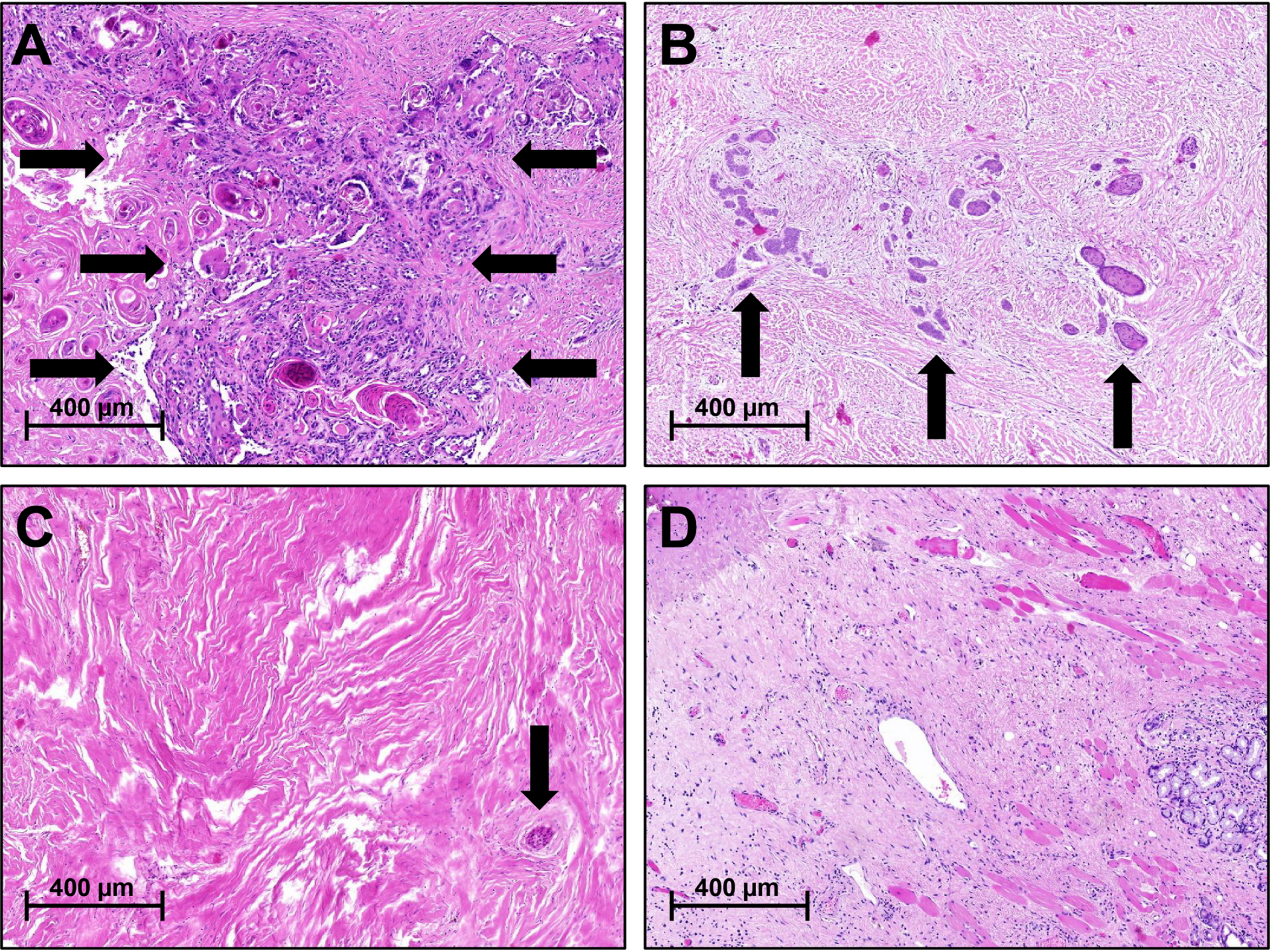
Pathological Response Patterns Following Neoadjuvant Chemoradiotherapy. **(A)** No/minimal tumor regression, vital tumor cells, and prominent keratin pearls; **(B)** Tumor fragmentation with increased amount of fibrous connective tissue with scattered groups of vital tumor cells; **(C)** Tumor shrinkage with a solitary group of vital tumor cells embedded in fibrous connective tissue; **(D)** Complete response with no vital tumor cells within fibrous connective tissue; salivary glands, and skeletal muscles located on the right.

Overall, a pCR (ypT0N0) in the intention-to-treat population was achieved in 7 (41%%, 95% CI: 18-67%) of the patients and in 44% (95% CI: 20-70%) of the patients who underwent surgery: ypT0 occurred in 8 (50%, 95% CI: 25-75%) and ypN0 in 13 (81%, 95% CI: 54-96%). In the majority of the patients with residual tumor, tumor fragmentation was found rather than tumor shrinkage. Exemplary images of tumor regression patterns are shown in **Figure 1**. Downsizing of the primary tumor of > 95% was evident in 88% (14/16, 95% CI: 68-98%) of the cases (**Table 2**).

### 3.3 Association of Diffusion-Weighted Magnetic-Resonance Imaging and Response to Chemoradiotherapy

**Figure 2:**
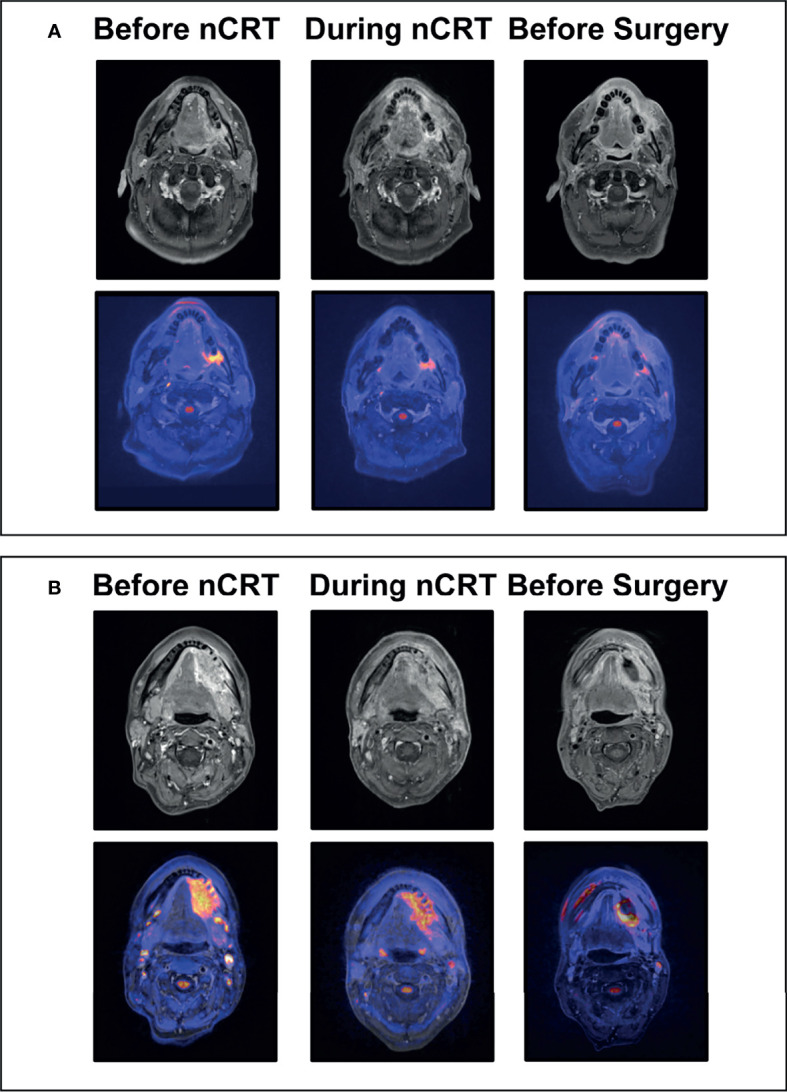
Exemplary MRI Images of Clinical Responses to Neoadjuvant Chemoradiotherapy. **(A)** Exemplary images of a 55-year old patient with left-sided squamous cell carcinoma of the oral cavity before and during chemoradiotherapy (day 15), and prior to surgery; The top row shows representative axial gadolineum-enhanced T1-weighted images with continuous decrease in size and contrast enhancement resulting in complete clinical response prior to surgery of the primary tumor at the left retromolar region; The bottom row shows corresponding fused diffusion-weighted - gadolineum-enhanced T1-weighted images with decreasing diffusion restriction of the tumor region resulting in complete clinical response prior to surgery. **(B)** Exemplary images of a 49-year old patient with left-sided squamous cell carcinoma of the oral cavity before and during chemoradiotherapy (day 15), and prior to surgery; The top row shows representative axial gadolineum-enhanced T1-weighted images with continuous decrease in size and contrast enhancement. Markable residual tumor with contrast enhancement at the left mandibular region prior to surgery; The bottom row shows corresponding fused diffusion-weighted - gadolineum-enhanced T1-weighted images with decreasing but residual diffusion restriction of the tumor region; nCRT, Neoadjuvant chemoradiotherapy.

**Figure 3:**
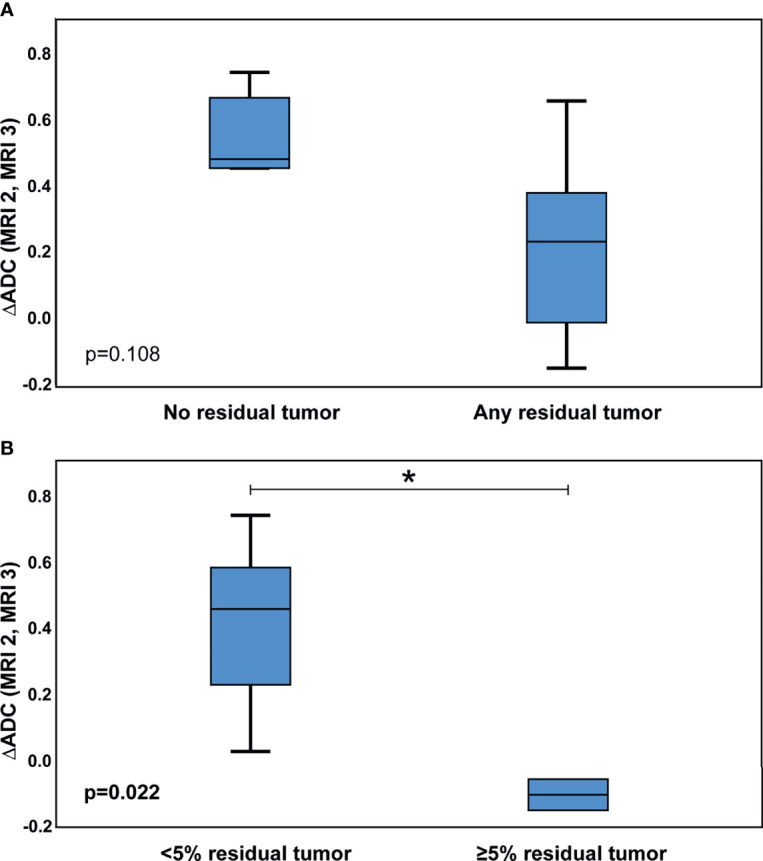
Association of Changes in ADC Signal Intensities with Pathological Tumor Response. Delta (Δ) in ADC signal intensities of MRI 2 and MRI 3 correlated with pathological response of the primary tumor: **(A)** ΔADC, complete response of the primary tumor vs. any residual primary tumor; **(B)** ΔADC, <5% residual primary tumor vs. ≥5% residual primary tumor; ADC, Apparent diffusion coefficient; MRI, Magnetic resonance imaging; p-values according to Mann-Whitney U test; *p-value < 0,05.

The test for inter-rater variability regarding the qualitative MRI evaluation showed high correlation between the two raters (kappa 0,809; p<0.001). Qualitative signal intensities changed significantly between MRI 1, MRI 2, and MRI 3 in diffusion-weighted and T1 + gadolinium series (p-values < 0.05). Exemplary, fused axial diffusion-weighted gadolineum-enhanced T1-weighted images are shown in **Figure 2**. Regarding quantitative analysis, signal intensities changed significantly when MRI 2 and MRI 3 were compared to MRI 1 in the ADC and diffusion-weighted series (p-values < 0.05), and between MRI 2 and MRI 3 in the T2 series (p=0.034) (**Supplementary Table 4**). Quantitative and qualitative changes in signal intensities were correlated with the pathological response of the primary tumor following CRT. Increased changes in the ADC signal intensity between MRI 2 and 3 were associated with < 5% residual tumor tissue (p=0.022) (**Figure 3**, **Supplementary Table 5**).

### 3.4 Association of Immunohistochemical Biomarkers in Pre-Treatment Tissue Specimens Imaging and Response to Chemoradiotherapy

**Figure 4:**
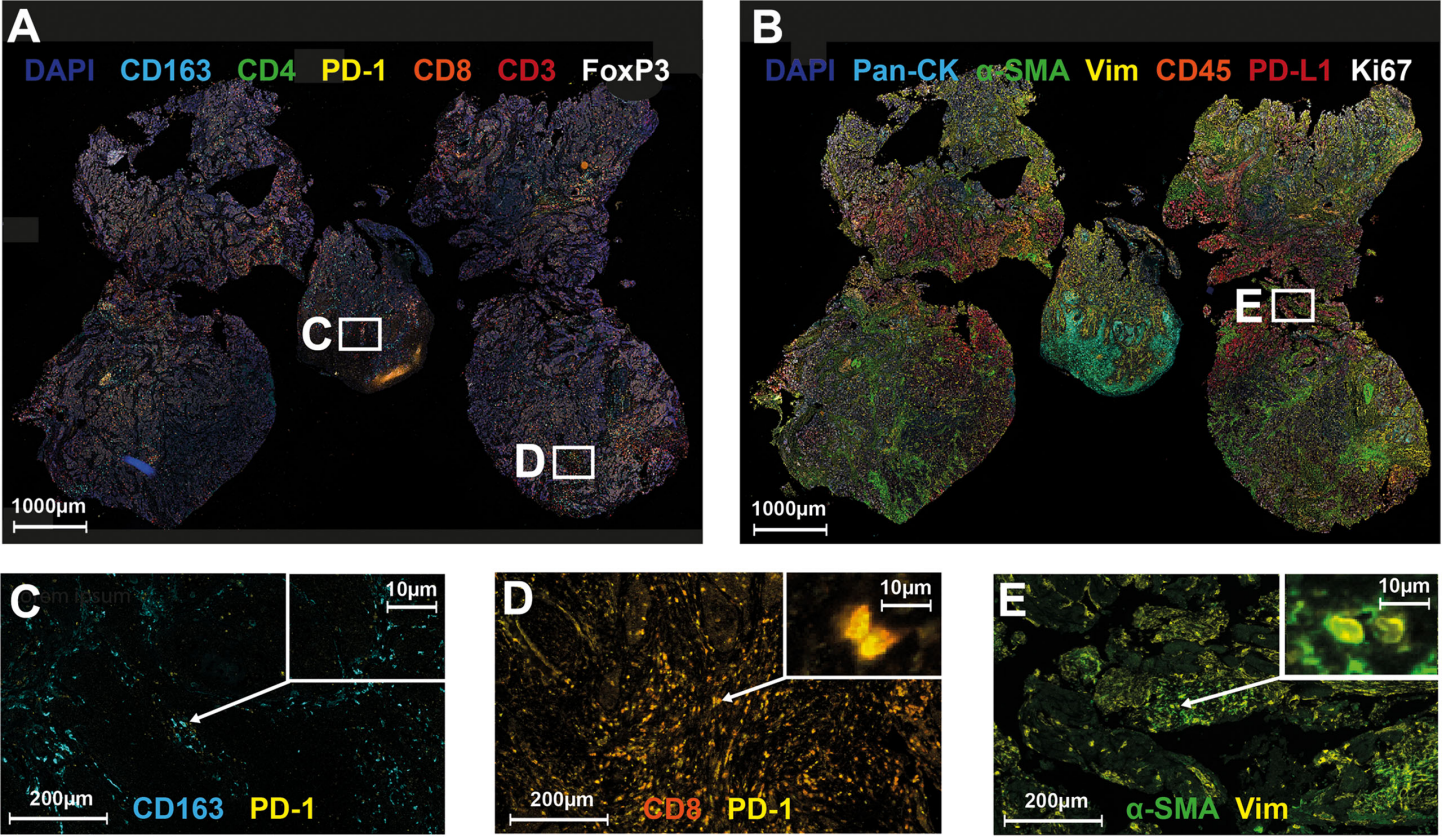
Multiplex Immunohistochemistry and Cell Types Associated with Poor Response to Neoadjuvant Chemoradiotherapy. Representative overview of the T-cell antibody panel **(A)** and the TME panel **(B)**, and exemplary images of cell types with association to tumor response to neoadjuvant chemoradiotherapy **(C–E)**. Nuclei were counterstained with DAPI (blue). **(A)** T-cell panel: CD163 (cyan), CD4 (green), PD-1 (yellow), CD8 (orange), CD3 (red), FoxP3 (white); **(B)** TME panel: Pan-CK (cyan), aSMA (green), Vimentin (yellow), CD45 (orange), PD-L1 (red), Ki67 (white); **(C)** PD-1 positive macrophage; **(D)** PD-1 positive cytotoxic T-cell; **(E)** Cancer-associated fibroblast.

To identify possible predictive markers for response to nCRT, the abundance of different cell populations was tested for their association with either pCR or ypT0 (**Table 3** and **Figure 4**). A higher abundance of PD1+ cytotoxic T-cells (p=0.012) and PD1+ macrophages (p=0.046) was associated with incomplete response of the primary tumor to nCRT (no ypT0). Further, an increased occurrence of PD1+ cytotoxic T-cells (p=0.036) and CAFs (p=0.036) was associated with incomplete tumor and or nodal response (no pCR).

**Table 3:** Association of pre-treatment immune cell infiltration and cells of the tumor microenvironment with response to neoadjuvant chemoradiotherapy.

Cell types	ypT0N0, n (%)			ypT0, n (%)		
Total n=16	ypT0N0	Rest	p	ypT0	Rest	p
**T-Helper Cells**						
Low	3 (37)	5 (63)		3 (37)	5 (63)	
High	4 (50)	4 (50)	0.614	5 (63)	3 (37)	0.317
**Exhausted T-Helper Cells**						
Low	4 (50)	4 (50)		5 (63)	3 (37)	
High	3 (37)	5 (63)	0.614	3 (37)	5 (63)	0.317
**Regulatory T-Cells**						
Low	4 (50)	4 (50)		5 (63)	3 (37)	
High	3 (37)	5 (63)	0.614	3 (37)	5 (63)	0.317
**Cytotoxic T-Cells**						
Low	3 (37)	5 (63)		4 (50)	4 (50)	
High	4 (50)	4 (50)	0.614	4 (50)	4 (50)	1.000
**PD1+ cytotoxic T-Cells**						
Low	6 (67)	3 (33)		7 (78)	2 (22)	
High	1 (14)	6 (86)	**0.036**	1 (14)	6 (86)	**0.012**
**Macrophages**						
Low	2 (25)	6 (75)		3 (37)	5 (63)	
High	5 (63)	3 (37)	0.131	5 (63)	3 (37)	0.317
**PD1+ macrophages**						
Low	5 (63)	3 (37)		6 (75)	2 (25)	
High	2 (25)	6 (75)	0.131	2 (25)	6 (75)	**0.046**
**Cancer-associated fibroblasts**						
Low	6 (67)	3 (33)		6 (67)	3 (33)	
High	1 (14)	6 (86)	**0.036**	2 (29)	5 (71)	0.131
**PD-L1+ Cancer-associated fibroblasts**						
Low	3 (37)	5 (63)		4 (50)	4 (50)	
High	4 (50)	4 (50)	0.614	4 (50)	4 (50)	1.000
**Immune Cells**						
Low	4 (44)	5 (56)		4 (44)	5 (56)	
High	3 (43)	4 (57)	0.949	4 (57)	3 (43)	0.614
**PD-L1+ Immune Cells**						
Low	3 (33)	6 (67)		4 (44)	5 (56)	
High	5 (57)	3 (43)	0.341	4 (57)	3 (43)	0.614
**Proliferating Immune Cells**						
Low	4 (40)	6 (60)		4 (50)	4 (50)	
High	3 (50)	3 (50)	0.696	3 (50)	3 (50)	1.000

P-values according to Pearson chi-squared test. Bold values indicate p-values <0.05.

## 4 Discussion

Only a limited number of studies have investigated nCRT for HNSCC to date. We present preliminary clinical and translational results of a single-arm, prospective trial utilizing neoadjuvant, concomitant IMRT-based CRT followed by radical surgery, and provide novel predictive biomarkers, such as immune cell infiltrates and diffusion weighted MRI imaging.

The use of nCRT is standard in different tumor entities, such as lung, esophageal and rectal cancer, with encouraging pCR rates and long-term oncologic outcomes (23–26). In HNSCC, primary surgery with risk-adapted adjuvant RT/CRT has been the standard of care for decades, but has never been tested against nCRT in a prospective, randomized trial (1, 2). A number of retrospective studies have investigated nCRT for HNSCC of different subsites with RT doses ranging from 20-50 Gy. Concomitant systemic therapy was mostly platinum-based with cumulative doses between 63–100 mg/m². The time interval from the end of CRT to surgery ranged between 1-6 weeks, resulting in pCR rates form 0-50%, and 5 years overall survival (OS) rates of 45-81% (**Supplementary Table 6**) (27–35). In 7 prospective, non-randomized trials, neoadjuvant RT doses of 40-50 Gy were applied with (n=6) or without (n=1) concomitant CTX. Again, CTX was mostly platinum-based with cumulative doses of 160-200 mg/m². Intervals from completion of RT/CRT to surgery ranged from 3-8 weeks with pCR rates from 13 to 75% (**Supplementary Table 7**) (36–44). A randomized study by Mohr et al. assigned 268 patients to surgery alone or nCRT with 36 Gy and concomitant cisplatin (12.5 mg, days 1-5), followed by radical surgery 10-14 days after CRT completion. In this study, nCRT resulted in pCR of the primary tumors in 37% of the patients, and less locoregional relapses occurred after 3 years (31% vs. 16%) (38). Yi et al. randomized patients to receive neoadjuvant RT (50 Gy) with or without concomitant cisplatin (cumulative 150 mg/m²). Following local restaging with CT/MRI and endoscopy, patients received completion CRT (total 70 Gy + cisplatin) for >80% clinical remission, followed by planned neck dissections for cN2-3 patients, or radical surgery after 6-8 weeks. Surprisingly, clinical response rates (64 vs. 70%) and pCR rates (27 vs. 43%) were lower following nCRT compared to neoadjuvant RT alone. However, local progression-free survival and OS were improved following nCRT versus neoadjuvant RT and surgery (44). Most of the patients included in the studies above would have received standard, adjuvant RT doses of 60-66 Gy resulting in disease-free survival rates of less than 50% at 5 years (1, 2). However, most of the above neoadjuvant data originate from the pre-IMRT era, where dose escalation was clearly associated with higher toxicity. Accordingly, a higher dose of 60 Gy was selected for this IMRT-based trial. Further, the cumulative doses of cisplatin in the older studies were mostly far less than the currently recommended ≥ 200mg/m² utilized in combination with 5-FU in this study (45). CTX consisting of cisplatin plus 5-FU is not the current international standard for HNSCC. However, in our department as in other German-speaking centers cisplatin (200mg/m² total) and 5-FU was the standard concomitant CTX regimen at the time the trial was designed. Furthermore, a parallel German multicentric phase III trial in the definitive CRT-setting, failed to demonstrate any benefit regarding survival or toxicity for a taxane/cisplatin combination compared to the cisplatin/5-FU regimen used in this trial, with the latter showing good 3 years OS rates of 65% (15).

The time interval between CRT and surgery was scheduled to be 6 to 8 weeks in this trial and therefore longer than in the majority of the prior trials. There is little experience regarding re-growth of HNSCC after neoadjuvant regimens in cases of delayed surgery. However, in other tumor entities treated with neoadjuvant CRT, such as rectal cancer or esophageal cancer, surgery is commonly performed 6-8 weeks after CRT completion in order to allow for prolonged tumor regression (23–26). Furthermore, in anal squamous cell carcinoma, a tumor entity with several biological parallels to HNSCC, it has been demonstrated that a final response evaluation should be performed 6 months after CRT (50-60 Gy) completion (46). Moreover, for primary CRT of HNSCC, tumor response also is only evaluated at 3 months following treatment and any residual tumor after 6 to 8 weeks after treatment is not necessarily considered as clonogenic (47). The feasibility of surgery and the frequency of postoperative complications were of special interest in this study. In all patients, complete tumor resections and adequate ND were possible. Surgical complications are frequently classified using a system introduced by Clavien and Dindo (16) which has been adapted for head and neck cancer as well (17, 48). McMahon et al. prospectively studied postoperative complications according to the CD system in 192 patients who underwent major head and neck surgeries with free flap repair. A total of 64% had any-grade complications with grade 3 or above occurring in 32% of the patients. Loss of flaps occurred in 3 patients (49). Peters et al. reported 60% overall complications from a cohort of 121 patients with more than half of them being major (grade 3-5) (50), and Grammatica et al. reported on 84 patients with 62% complication rate with 31% of grade 3 and higher (51). In the present trial, a total of 68% of the patients suffered from post-operative complications and 56% had grade 3 complications. One loss of flap and no grade 4-5 toxicities occurred in the context of surgery. Overall complications did not occur more frequently here in comparison with the rare literature on this topic.

Within our study, extended tumor regression analysis besides the general TNM classification was utilized to more precisely assess response patterns. Braun et al. developed a tumor regression grading (TRG) for HNSCC on the basis of the percentage of vital residual tumor cells (18). Analogous to a recent system introduced by Nagtegaal et al. for rectal cancer, regression patterns in this study were also distinguished between tumor fragmentation and shrinkage (19). Tumors without complete response to nCRT more likely showed fragmentation (n=5) instead of shrinkage (n=3). Prediction of tumor shrinkage following nCRT rather than tumor fragmentation would be of great value for clinicians to possibly reduce the extent of surgery, but higher patient numbers are needed to address this topic. To date, surgery for HNSCC should be performed within the initial tumor margins due to potential tumor fragmentation. Tumor fragmentation following nCRT in HNSCC possibly reflects radio-resistant, hypoxic or immune-privileged tumor subareas, and has been associated with tumor recurrence by Kiong et al. (52). This hypothesis is further supported by the following immunological findings: Multiplex IHC in this study showed that higher abundances of PD1+ cytotoxic T-cells, PD1+ macrophages, and CAFs were associated with incomplete response to nCRT. The prognostic value of immune cell infiltrates and the TME composition have been extensively studied in HNSCC within the last years (53, 54). CD8+ tumor-infiltrating lymphocytes (TILs) were shown to be prognostic factors associated with improved outcome following primary or adjuvant CRT in single- and multicenter cohorts (55, 56). On the other hand, PD1 is a prominent marker of T-cell exhaustion and inhibits anti-tumor T-cell response (57). M2-polarized (CD163+), tumor-associated macrophages (TAMs) promote tumor growth and spread (58). PD1+ expression in TAMs negatively correlates with their phagocytic effects against tumor cells (59) and high abundances of PD1+ TAMs were associated with poor outcome in gastric and muscle-invasive bladder cancer, yet (60, 61). CAFs were reported to play a key role in tumor progression by secretion of growth factors and cytokines, and high αSMA levels in OCC were associated with impaired prognosis (62–65). Taken together, in patients with incomplete response to nCRT, the tumor and its microenvironment were defined by immunosuppressive stimuli and exhausted immune effector cells.

Finally, we identified an association of changes in ADC signal intensities with response to nCRT. Previously, Kato et al. identified correlations of tumor regression according to RECIST (Response evaluation criteria in solid tumors) with ADC and diffusion-weighted signal intensities in 28 HNSCC patients treated with neoadjuvant CRT, RT, or CTX (20). Median RT dose applied was 30 Gy. Imaging was performed before and after neoadjuvant treatment. To the best of our knowledge, no other study has analyzed early and late responses to nCRT *via* DW-MRI in HNSCC to predict pathological tumor response. So far, DW-MRI studies for HNSCC have mostly focused on early response prediction either during or after definitive CRT (66, 67). Kim et al. performed DW-MRI on 40 patients undergoing primary CRT for HNSCC before, during, and after therapy. Complete therapy responders showed an early increase in ADC intensity (p<0.01) (68). Further studies found high pretreatment ADC intensities to be associated with poor outcome in HNSCC (69, 70). Besides these encouraging results, the DW-MRI evaluation procedures to assess response to therapy have differed greatly between the previous studies and standardized evaluation protocols to improve comparability were not yet established.

We acknowledge several limitations of this study: First, the sample size is limited and allows only preliminary and exploratory hypotheses regarding the predictive biomarkers assessed. Second, the unicentric character of the study warrants caution regarding generalization of the results. Third, this interim analysis was not planned according to the study protocol. Finally, some surgical techniques and DW-MRI quantification are not completely standardized yet, which might affect interpretability. Nevertheless, immunological and radiological biomarkers were correlated with pathological responses to neoadjuvant CRT for this tumor entity for the first time.

## 5 Conclusion

Neoadjuvant chemoradiotherapy for locally advanced oral cavity cancer followed by radical surgery is feasible and shows high response rates. Emerging biomarkers such as diffusion-weighted magnetic resonance imaging signal intensities, tumor immune cell infiltrates, and the tumor microenvironment are of great interest with potential predictive value regarding response following neoadjuvant treatment. Ultimately, future patient selection for organ preservation could be based on these factors following randomized, controlled trials.

## Data Availability Statement

The data presented in this study are available on request from the corresponding author. Requests to access the datasets should be directed to Jens von der Grün, jens.vondergruen@kgu.de.

## Ethics Statement

The studies involving human participants were reviewed and approved by Ethics committee of the Goethe-University Frankfurt, Frankfurt, Germany; Approval No.: 208/12. The patients/participants provided their written informed consent to participate in this study.

## Author Contributions

Conceptualization, JG, CR, SG, and PB. Methodology, JG, NF, CR, SG, and PB. Data acquisition, JG, RW, IB, PW, KB, AW, I-MK, CB, CI, PT, AT, SG, and PB. Analysis, JG, RW, IB, DM, FR, KB, AW, I-MK, CB, NF, CI, PT, CR, AT, SG, and PB. Investigation, JG, IB, CB, PT, CR, SG, and PB. Writing—original draft preparation, JG, NF, RW, AW, I-MK, FR, IB, CR, SG, and PB. Writing—review and editing, JG, RW, IB, DM, FR, PW, KB, AW, I-MK, CB, AT, NF, CI, PT, CR, SG, and PB. Visualization, JG, IB, and DM. Project administration, JG, CR, SG, and PB. Funding acquisition, PB. All authors have read and agreed to the final version of the manuscript.

## Funding

The study was partially funded by a research grant received by Panagiotis Balermpas for the INVERT trial by the Clinical Trial Center Network of the University Cancer Center Frankfurt, 2012.

## Conflict of Interest

PW has received consulting fees and honoraria (private/institutional) for lectures by Bayer, Janssen-Cilag, Novartis, Roche, MSD, Astellas Pharma, Bristol-Myers Squibb, Thermo Fisher Scientific, Molecular Health, Sophia Genetics, Qiagen, Eli Lilly, Myriad, Hedera Dx, and Astra Zeneca.

The remaining authors declare that the research was conducted in the absence of any commercial or financial relationships that could be construed as a potential conflict of interest.

## Publisher’s Note

All claims expressed in this article are solely those of the authors and do not necessarily represent those of their affiliated organizations, or those of the publisher, the editors and the reviewers. Any product that may be evaluated in this article, or claim that may be made by its manufacturer, is not guaranteed or endorsed by the publisher.
