# Supplementary material for: Neoadjuvant Chemoradiotherapy for Oral Cavity Cancer: Predictive Factors for Response and Interim Analysis of the Prospective INVERT-Trial
Source: Front Oncol. 2022 Mar 24;12:817692. doi: 10.3389/fonc.2022.817692 (PMC8988145; doi:10.3389/fonc.2022.817692)
Supplement: Supplementary file 1 [file DataSheet_1.zip › Supplementary figure 2.PDF]

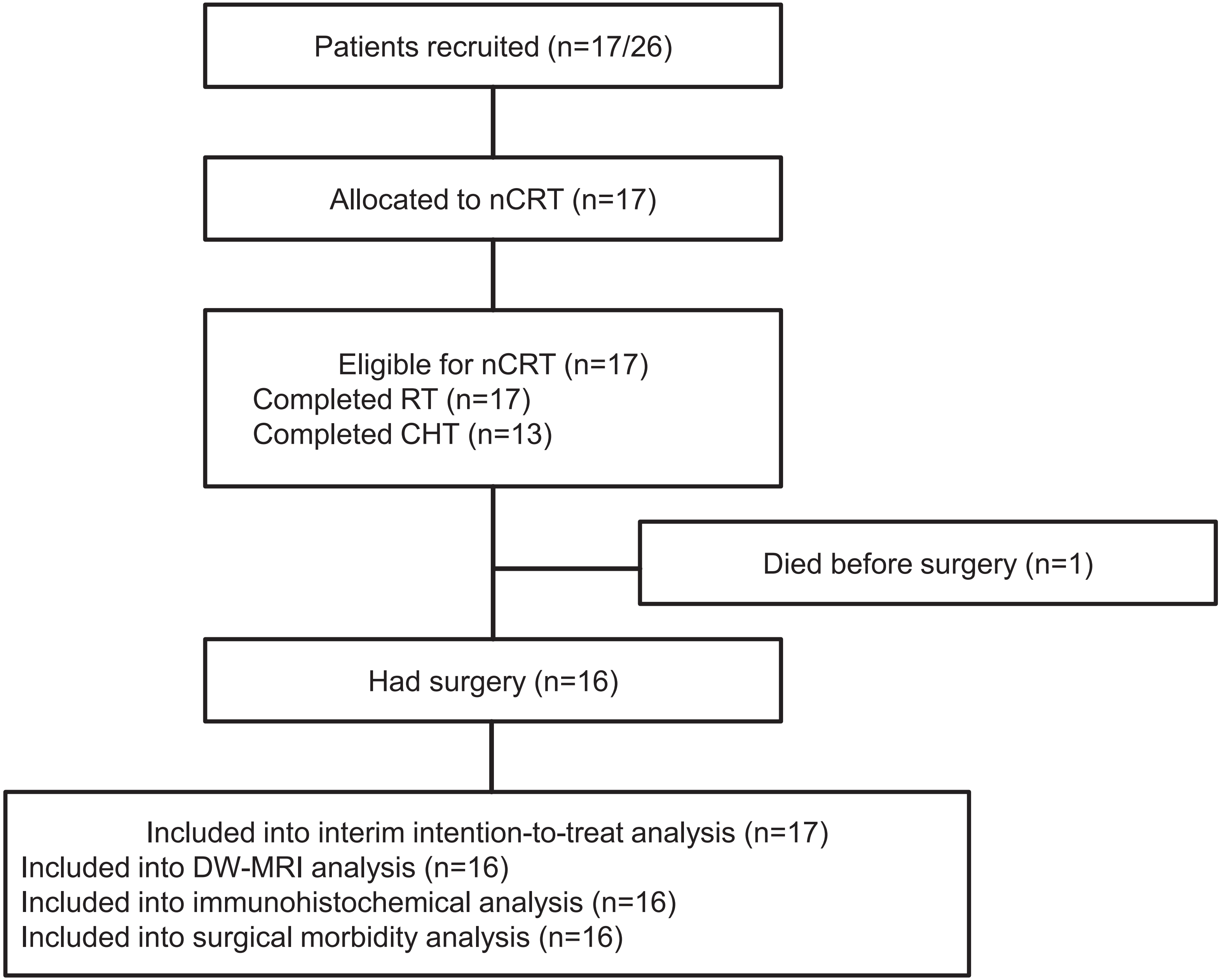

Supplementary figure 2: CONSORT Diagram of the INVERT Trial. Abbreviations: nCRT, Neoadjuvant chemoradiotherapy; DW-MRI, Diffusion-weighted magnetic resonance imaging.
