# Supplementary material for: Neoadjuvant Chemoradiotherapy for Oral Cavity Cancer: Predictive Factors for Response and Interim Analysis of the Prospective INVERT-Trial
Source: Front Oncol. 2022 Mar 24;12:817692. doi: 10.3389/fonc.2022.817692 (PMC8988145; doi:10.3389/fonc.2022.817692)
Supplement: Supplementary file 1 [file DataSheet_1.zip › Supplementary tables.docx]

**Supplementary table 1: INVERT Study Protocol Synopsis.**

| **Title of Study** | **Phase II study to assess the results of intensity-mudulated radiotherapy based neoadjuvant chemoradiotherapy with local dose escalation, followed by radical resection for squamous cell carcinoma of the head and neck (INVERT).** |
| --- | --- |
| **Study Chairman** | Prof. Dr. Dr. Dr. Shahram Ghanaati |
| **Rationale** | The benefit of simultaneous radiochemotherapy in the treatment of locally advanced squamous cell carcinoma of the head and neck has been confirmed by several studies, both in the adjuvant (Bernier et al (EORTC 22931), Cooper et al (RTOG 9501) and definitive situation (Adelstein, JCO, 2003; Brizel, NEJM, 1998, Budach, JCO, 2005).  Only one prospective randomized study (Mohr, Int J Oral Maxillofac Surg, 1994) and several smaller studies of limited evidence level exist so far regarding neoadjuvant chemoradiotherapy of head and neck cancer. The study by Mohr et al. showed promising results and survival data were by no means inferior to adjuvant or definitive therapy, although the technique and dosage of radiotherapy are considered outdated by today's standards.  Experience in other tumor entities, such as rectal cancer, showed that better outcomes are possible by this modality (Sauer et al., NEJM, 2004), so that nowadays neoadjuvant therapy is recommended by the guidelines for these tumors. Many reasons could potentially contribute to an improved prognosis in head and neck patients, including improved patient compliance to radiochemotherapy, early systemic therapy, as complication-related delays are often expected postoperatively, and better perfusion and oxygenation in the unoperated tissue, which is associated with increased radiosensitivity.  By integrating modern irradiation techniques, such as intensity-modulated radiotherapy (IMRT), a more precise target volume detection as well as a small volume dose saturation can be achieved with relatively few complications, so that possibly even better results than in the studies published so far using 3D techniques would be possible. The rate of side effects could be reduced subjectively and objectively by the use of IMRT. Data on this in the definitive and adjuvant situation exist in various publications (e.g. Chao, Int J Radiat Oncol Biol Phys., 2001). |
| **Study Type and Study Design** | Prospective, single-arm, single-center phase II study |
| **Primary Objective and Endpoint** | - Documentation and evaluation of the effectiveness and toxicity of radiochemotherapy followed by standard surgery. - Pathological complete response (pCR) |
| **Secondary Endpoint** | - Postoperative complications - Quality of life - 2 years Overall and Progression-free survival - 2 years locoregional control-rate - Translational research program (i.e. immunohistochemical biomarkers) |
| **Inclusion Criteria** | - Male and female patients with histologically confirmed diagnosis of squamous cell carcinoma localized in the oral cavity, or oropharynx - Staging requirements: High-resolution MR imaging is mandatory. MRI-defined inclusion criteria: at least one of the following risk constellations: - cT3 /cT4 - cT1/cT2 with cN2-3 - cM0 according to computed tomography (CT) of thorax and abdomen - Aged at least 18 years. No upper age limit. - WHO/ECOG Performance Status ≤2 - Adequate haematological, hepatic, renal and metabolic function parameters: - Leukocytes ≥ 3.000/mm^3, ANC ≥ 1.500/mm^3, platelets ≥ 100.000/mm^3, Hb > 9 g/dl - Serum creatinine ≤ 1.5 x upper limit of normal - Bilirubin ≤ 2.0 mg/dl, SGOT-SGPT, and AP ≤ 3 x upper limit of normal - Informed consent of the patient |
| **Exclusion Criteria** | - Distant metastases (to be excluded by CT scan of the thorax and abdomen) - Prior radiotherapy of the head and neck region - Prior major surgeries of the head and neck region - Subject pregnant or breast feeding, or planning to become pregnant within 6 months after the end of treatment - Subject (male or female) is not willing to use highly effective methods of contraception (per institutional standard) during treatment and for 6 months (male or female) after the end of treatment (adequate: oral contraceptives, intrauterine device or barrier method in conjunction with spermicidal jelly). - Previous or current drug or alcohol abuse - Other concomitant antineoplastic therapy - Serious concurrent diseases, including neurologic or psychiatric disorders (incl. dementia and uncontrolled seizures), active, uncontrolled infections, active, disseminated coagulation disorder - Clinically significant cardiovascular disease in (incl. myocardial infarction, unstable angina, symptomatic congestive heart failure, serious uncontrolled cardiac arrhythmia) ≤ 6 months before enrollment - Prior or concurrent malignancy ≤ 5 years prior to enrolment in study (Exception: non-melanoma skin cancer or cervical carcinoma FIGO stage 0-1), if the patient is continuously disease-free - Known allergic reactions on study medication - Known dihydropyrimidine dehydrogenase deficiency - Psychological, familial, sociological or geographical condition potentially hampering compliance with the study protocol and follow-up schedule (these conditions should be discussed with the patient before registration in the trial) |
| **Treatment** | Diagnostics, radiotherapy, chemotherapy and surgery are performed as part of standard care. To facilitate implementation, the essential components of the treatment concept and the expected side effects are summarized in the protocol. The decision on the choice of therapy and its implementation are the responsibilities of the treating physician.  Initiation of radiotherapy within approximately 2 weeks after histologic confirmation and tattooing of primarius borders, quality of life assessment, and staging with diffusion-weighted MRI of the neck, and CT thorax/abdomen. Diffusion-weighted MRI of the neck is further performed after 10 fractions of radiotherapy to assess early responses and prior to surgery.  Boost volume definition follows interdisciplinary discussion, and adresses, according to the surgeon's assessment, the region with the greatest resection difficulty.  Neoadjuvant radiotherpy consists of 60.0/54.9/50.1 Gy in 30 fractions, applied to the primary tumor region, involved/high risk neck levels, and the elective neck levels according to current guidelines, respectively. Intensity-modulated radiotherapy with a simultaneously integrated boost concept is used. Two cycles of chemotherapy are applied on days 1–5, and 29–33 of the radiotherapy consisting of 5-fluorouracil (5FU) (600 mg/m² per day) as a continuous 120-h intravenous infusion, and cisplatin (20 mg/m² per day) as short intravenous infusion.  Radical surgery following chemoradiotherapy is performed according to the initial extension of the primary tumor as marked by pre-treatment tattooing. Elective neck dissection is performed according to pre-treatment staging information. Elective, ipsilateral supraomohyoid neck dissection (SOHND) is conducted for clinically negative neck nodes (cN0), and will be extended to the neck levels I-V for pathologically positive nodes. In these cases and for tumors crossing midline, contra-lateral SOHND is performed and also extended to the neck levels I-V for positive, contra-lateral nodes.  Follow-up consists of clinical controls every 4-8 weeks and MRI/CT neck, and quality of life assessment every 3 months, CT thorax/abdomen every 6 months for the first 2 years. |
| **Translational Research** | An extensive translational research program is implemented in order to further refine prognostic and predictive profiling (i.e. immunohistochemical biomarkers). |
| **Sample Size and Justification** | The primary clinical objective of this pilot study is to estimate the pCR rate and to calculate the corresponding 95% confidence interval. In order for the overall statistical length to be less than 40%, data from a total of n=26 patients must be available for analysis (exact Clopper-Pearson calculation). Since the primary endpoint of pCR is achieved after surgery, we expect only a small drop out of at most 5%, resulting in a number of 28 patients to be recruited. |
| **Biostatistical Methods** | The results collected in the study will provide the data basis for planning further interventional studies.  All primary analyses will follow the ITT principle, i.e. all randomized patients will be included in the analyses and in the treatment groups they were randomized to. For the primary efficacy outcome, the proportions of patients with pCR will be reported with 95% confidence intervals and the treatment groups will be compared in a logistic regression with pCR as dependent variable and independent variables (i.e. tumor and treatment characteristics) as factors.  The assumed probability for pCR is at least 33% and the overall length is less than 40% (i.e., 20% down and up). Time-to-event data such as progression-free survival and overall survival are presented as Kaplan-Meyer curves with 95% confidence intervals. Drop out will be treated as independent right censoring. |
| **Study Duration** | Start of recruitment: 05/2013  Planned termination of recruitment: 12/2023  Planned termination of follow-up: 12/2025  Final study report: 06/2026 |

**Supplementary table 2: Acute Adverse Effects in Patients Receiving Neoadjuvant Chemoradiotherapy.** *National Cancer Institute Common Terminology Criteria for Adverse Events version 5.0. ^#^One patient with comorbidities died from pneumonia ten weeks after completion of chemoradiotherapy without surgery following refusal of hospital admission; Abbreviations: NA, Not applicable.

| Toxicity* | Grade 1-2 | Grade 3 | Grade 4 | Grade 5 |
| --- | --- | --- | --- | --- |
|  | **n (%)** | | | |
| Hematologic |  |  |  |  |
| Leukocytes | 9 (53) | 5 (29) | 0 (0) | 0 (0) |
| Hemoglobin | 7 (41) | 0 (0) | 0 (0) | 0 (0) |
| Platelets | 9 (53) | 0 (0) | 0 (0) | 0 (0) |
| Infection | 6 (35) | 1 (6) | 0 (0) | 1^#^ (6) |
| Gastrointestinal |  |  |  |  |
| Nausea | 11 (65) | 0 (0) | NA | NA |
| Vomiting | 2 (12) | 0 (0) | 0 (0) | 0 (0) |
| Obstipation | 12 (71) | 0 (0) | 0 (0) | 0 (0) |
| Genitourinary |  |  |  |  |
| Creatinine | 8 (47) | 0 (0) | 0 (0) | NA |
| Cardiac |  |  |  |  |
| Arrythmia | 0 (0) | 0 (0) | 0 (0) | 0 (0) |
| Ischemia | 0 (0) | 0 (0) | 0 (0) | 0 (0) |
| Hypertension | 8 (47) | 6 (35) | 0 (0) | 0 (0) |
| Other |  |  |  |  |
| Pain | 11 (65) | 4 (24) | NA | NA |
| Dysphagia | 11 (65) | 4 (24) | 0 (0) | 0 (0) |
| Mucositis oral | 10 (59) | 7 (41) | 0 (0) | 0 (0) |
| Radiation dermatitis | 15 (88) | 2 (12) | 0 (0) | NA |
| Weight loss | 7 (41) | 0 (0) | NA | NA |

**Supplementary table 3:** **Treatment Compliance with Neoadjuvant Chemoradiotherapy.** Abbreviations: 5FU, 5-fluorouracil; RT, radiotherapy; PEG , Percutaneous endoscopic gastrostomy.

| Compliance with neoadjuvant chemoradiotherapy | n (%) |
| --- | --- |
| Total | **17 (100)** |
| Radiotherapy |  |
| Without radiotherapy interruptions | 17 (100) |
| With radiotherapy interruptions | 0 (0) |
| Prescribed chemotherapy |  |
| Cisplatin/5FU | 13 (76) |
| Cisplatin | 3 (18) |
| Carboplatin/5FU | 1 (6) |
| Chemotherapy completion |  |
| 100% of planned dose | 13 (76) |
| >50% of planned dose | 4 (24) |
| Prophylactic PEG feeding tube |  |
| Yes | 17 (100) |
| No | 0 (0) |

**Supplementary table 4: Changes in MRI Signal Intensities in Patients Receiving Neoadjuvant Chemoradiotherapy.** Abbreviations: MRI, Magnetic resonance imaging; ADC, Apparent diffusion coefficient; p-values according to Wilcoxon test.

| MRI series | p-values | | |
| --- | --- | --- | --- |
| Total n=16 | **MRI 1 vs. MRI 3** | **MRI 1 vs. MRI 2** | **MRI 2 vs. MRI 3** |
| Quantitative assessment |  |  |  |
| ADC | **0.001** | **0.001** | 0.245 |
| Diffusion-weighted | **0.001** | **0.003** | 0.056 |
| T2 | 0.717 | 0.063 | **0.034** |
| T1 + gadolinium | 0.600 | 0.161 | 0.484 |
| Qualitative assessment |  |  |  |
| Diffusion-weighted | **0.001** | **0.002** | **0.004** |
| T2 | 0.470 | 1.000 | 0.366 |
| T1 + gadolinium | **0.003** | **0.014** | **0.046** |

**Supplementary table 5:** **Correlation of Changes in MRI Signal Intensities with Tumor Regression Following Neoadjuvant Chemoradiotherapy.** Abbreviations: MRI, Magnetic resonance imaging; ADC, Apparent diffusion coefficient; * response of the primary tumor; p-values according to Mann-Whitney U test.

| MRI series | p-values | | |
| --- | --- | --- | --- |
| Total n=16 | **∆(MRI 1, MRI 3)** | **∆(MRI 1, MRI 2)** | **∆(MRI 2, MRI 3)** |
|  | **Correlation of ∆MRI with <5% residual tumor* vs. rest** | | |
| Quantitative assessment |  |  |  |
| ADC | 0.114 | 0.933 | **0.022** |
| Diffusion-weighted | 0.171 | 0.571 | 0.088 |
| T2 | 0.417 | 0.933 | 0.700 |
| T1 + gadolinium | 0.154 | 0.750 | 0.250 |
| Qualitative assessment |  |  |  |
| Diffusion-weighted | 0.381 | 0.686 | 0.440 |
| T2 | 0.417 | 0.267 | 0.817 |
| T1 + gadolinium | 0.615 | 1.000 | 0.500 |
|  | **Correlation of ∆MRI with complete response* vs. rest** | | |
| Quantitative assessment |  |  |  |
| ADC | 0.094 | 0.281 | 0.108 |
| Diffusion-weighted | 0.463 | 0.72 | 0.852 |
| T2 | 0.234 | 0.382 | 0.798 |
| T1 + gadolinium | 0.181 | 0.114 | 1.000 |
| Qualitative assessment |  |  |  |
| Diffusion-weighted | 0.072 | 0.121 | 0.755 |
| T2 | 0.161 | 0.328 | 0.505 |
| T1 + gadolinium | 0.138 | 0.250 | 0.571 |

**Supplementary table 6: Retrospective Studies Investigating Neoadjuvant Radiotherapy for Locally Advanced Head and Neck Cancer.** Abbreviations: N, Number of patients; CTX, Chemotherapy; S, Surgery; pCR, Pathological complete response; MMC, Mitomycin C; 5FU, 5-fluorouracil; Cis, Cisplatin; Carbo, Carboplatin; OS, Overall survival; DFS, Disease-free survival; LCR, Local control rate; DSS, Disease-specific survival.

| First author (year) | N | Dose (Gy) | Concurrent CTX | Time to S  (weeks) | pCR (%) | Outcome |
| --- | --- | --- | --- | --- | --- | --- |
| Dobrowsky (1991) | 70 | 50 | MMC 15mg/m² d1  5FU 750mg/m² d1-5 | 3-5 | 49 | 5yrs OS: 61% |
| Kirita  (1999) | 48 | 40 | Cis 15mg/m² d1-3,22-24; Peplomycin 5mg d4-7, 25- 28 | 2-6 | 50 | 5yrs OS: 81%  5yrs DFS: 85% |
| Koelbl  (2001) | 88 | 40 | Cis 12.5-20mg/m² d1-5  Carbo 60mg/m² d1-5 | 3 | 33 | 5yrs OS: 63%  5yrs LCR: 79% |
| Freier  (2008) | 207 | 40 | Cis 12.5mg/m² d1-5 | 3-6 | 24 | 5yrs OS: 50%  5yrs DFS: 70% |
| Eich  (2008) | 134 | 39.6 | Carbo 70mg/m² d1-5 | 2-4 | 14 | 5yrs OS: 45%  5yrs DFS: 59% |
| Driemel  (2009) | 228 | 40 | Cis 20mg/m² d1-5 | 1-2 | 22 | 5yrs DSS: 76% |
| Klug  (2009) | 276 | 50 | MMC 15mg/m² d1  5FU 750mg/m² d1-5 | 4-6 | 43 | 5yrs OS: 54%  5yrs LCR: 70% |
| Mücke  (2011) | 407 | 20 | Cis 12.5mg/m² d1-5 | 3-6 | 0 | 5yrs OS: 69% |
| Kreppel  (2013) | 139 | 39.6 | Carbo 60mg/m² d1-5 | NA | 15 | 5yrs OS: 45% |

**Supplementary table 7: Prospective Studies Investigating Neoadjuvant Radiotherapy for Locally Advanced Head and Neck Cancer.** Abbreviations: N, Number of patients; RCT, Randomized controlled trial; NRT, Non-randomized trial; SA, Single-arm trial; OCC, Oral cavity cancer; CRT, Chemoradiotherapy; RT, Radiotherapy; CTX, Chemotherapy; S, Surgery; pCR, Pathological complete response; cCR, Clinical complete response; T, Primary tumor; 5FU, 5-fluorouracil; Cis, Cisplatin; Carbo, Carboplatin; S-1, Tegafur + prodrug of 5-fluorouracil + 5-Chloro-2-4-Dihydroxypyridine; OS, Overall survival; DFS, Disease-free survival; LCR, Local control rate; LRR, Local recurrence rate; DSS, Disease-specific survival; Mo, Months; mFU, Median follow-up; NR, Not reported; ND, Neck dissection.

| First author (year) | N | Design,  Primary tumor region | Treatment | Time to S  (weeks) | pCR / cCR (%) | Outcome |
| --- | --- | --- | --- | --- | --- | --- |
| Tupchong  (1991) | 354 | RCT,  mixed | A: S + RT(60Gy) vs.  B: RT(50Gy) + S vs.  [C: RT(70Gy) + salvage S] | 4-6 | NR | 4yrs OS: A:38%, B:33%  5yrs LCR: A:65%, B:48% |
| Slotman  (1992) | 53 | SA,  mixed | CRT(45Gy, Cis) + S + CTX(Cis) | 4-8 | cCR T: 75% | 5yrs OS: 43% |
| Mohr  (1994) | 268 | RCT, mixed | A: S vs.  B: CRT(36Gy, Cis) + S | 1-2 | pCR T: 37% | 3yrs LRR: A:31%, B:16% |
| Glicksman  (1994) | 101 | NRT,  mixed | CRT(45Gy, Cis +  A: RT(27Gy, for cCR) vs. B: S(no cCR) | 4 | cCR T: 66% | 5yrs OS: A:40%, B:55% |
| Wanebo  (2001) | 43 | NRT,  mixed | CRT(45Gy, Carbo/Taxol +  A: RT(27Gy) + ND (for pCR T) vs.  B: S | 1-2 | pCR T: 66% | 50mo mFU PFS: A+B: 65%  4yrs OS: A+B:68% |
| Keßler  (2007) | 74 | SA, OCC | CRT(50,4Gy, Cis/5FU) + S | NR | pCR: 38% | 5yrs OS: cT3:68%, cT4:51% |
| Eckardt  (2007) | 56 | SA,  mixed | CRT(40Gy, Carbo/Taxol) + S | 3-4 | pCR: 58% | 7yrs OS: 53%  7yrs LCR: 84% |
| Harada  (2013) | 39 | SA,  OCC | CRT(40Gy, S-1) + S | 3 | cCR T: 13% | 5yrs OS: 79%  5yrs LCR: 92% |
| Yi  (2017) | 222 | RCT,  mixed | A: CRT(50Gy, Cis) vs.  B: RT(50Gy)  Restaging:  ≥80% cCR T: CRT(20Gy, Cis) vs.  <80% cCR T: S | 6-8 | pCR after RT +S: 43%  pCR after CRT + S: 27% | 5yrs OS: A:54%, B:39%  5yrs LCR: A:70%, B:62% |
